# Supplementary material for: Unveiling the electronic transformations in the semi-metallic correlated-electron transitional oxide Mo8O23
Source: Sci Rep. 2019 Nov 4;9:15959. doi: 10.1038/s41598-019-52231-4 (PMC6828745; doi:10.1038/s41598-019-52231-4)
Supplement: Supplementary file 1 — Supplementary Information [file 41598_2019_52231_MOESM1_ESM.pdf]

# Supplementary Information for the article "Unveiling the electronic transformations in the semi-metallic correlated-electron transitional oxide $Mo_8O_{23}$"

V. Nasretdinova,[1,*] Ya. A. Gerasimenko,[1,2] J. Mravlje,[2] G. Gatti,[3] P. Sutar,[2] D. Svetin,[1,2] A. Meden,[4] V. Kabanov,[2] A. Yu. Kuntsevich,[5,6] M. Grioni,[3] and D. Mihailovic[1,2,7]

[1] *Center of Excellence on Nanoscience and Nanotechnology Nanocenter (CENN Nanocenter), Jamova 39, 1000 Ljubljana, Slovenia*
[2] *Jozef Stefan Institute, Jamova 39, 1000 Ljubljana, Slovenia*
[3] *Institute of Physics, EPFL, Lausanne, Switzerland*
[4] *Faculty of Chemistry and Chemical Technology, University of Ljubljana, Večna Pot 113, 1000 Ljubljana, Slovenia*
[5] *P. N. Lebedev Physical Institute of the Russian Academy of Sciences, 119991 Moscow, Russia*
[6] *National Research University Higher School of Economics, Moscow 101000, Russia*
[7] *Faculty of Mathematics and Physics, University of Ljubljana, Jadranska 19, 1000 Ljubljana, Slovenia*

## SUPPLEMENTARY NOTE 1: DFT-NESTING — SUSCEPTIBILITIES CALCULATION

In the susceptibility calculation we evaluated Eq. 1 by retaining sum over the band-indexes only for the two bands that are closest to the Fermi energy at temperature $T = 350\,\mathrm{K}$ in the high-temperature structure:

$$\chi(\mathbf{q}) = \sum_{\mathbf{k}\nu,\nu'} M(\mathbf{k}\nu, (\mathbf{k}+\mathbf{q})\nu') \frac{f(\epsilon_{k,\nu}) - f(\epsilon_{\mathbf{k+q},\nu'})}{\epsilon_{k,\nu} - \epsilon_{\mathbf{k+q},\nu'}} \tag{1}$$

where $\nu, \nu'$ are the band indexes, $f(\epsilon)$ are the Fermi functions and $\epsilon_{\mathbf{k}\nu}$ the band energies in the approximation $M(\mathbf{k}\nu, (\mathbf{k}+\mathbf{q})\nu') = |\langle \mathbf{k}\nu | e^{i\mathbf{q}r} | (\mathbf{k}+\mathbf{q})\nu' \rangle|^2 = \delta_{\nu,\nu'}$

If other bands are included in the evaluation (not shown), the value of the susceptibility increases. The unoccupied electronic bands have very little dispersion in the $k_x, k_z$ plane and hence to a good approximation simply add up to the signal shown on the Figure 4(d,e), but do not bring in a new dependence on the in-plane momentum.

## SUPPLEMENTARY NOTE 2: PMR IN THE TWO-LIQUID MODEL

We explain the PMR with the two-liquid model: total conductivity is the sum of contributions of at least two groups of carriers with different mobility. The magnetoresistance should be maximal, when the conductivities of these two groups are the closest. At high temperatures the carrier density and hence conductivity of first group is much larger than of the other one, hence it dominates in transport and PMR vanishes. As temperature decreases, the density drops, conductivities of different groups become comparable and PMR reaches its maximum. Finally, at low $T$, first group becomes empty, the system turns back to being single-group and PMR becomes vanishingly small again.

## SUPPLEMENTARY DISCUSSION 1: DIRAC CROSSING AT ZONE BOUNDARY

Dirac crossing (Dirac nodal line) is expected by DFT to become gapped for the low-T structure, but the gap is not resolved in the spectroscopic measurements. The Dirac nodal line is present in the high-T structure at the Brillouin zone boundary at $E - E_F \sim 0.7\,\mathrm{eV}$ (cf. Fig. 3 in main text). Its peculiar nature stems from the fractional translational symmetry[1, 2] – the $c$-glide plane that yields non-symmorphic symmetry of both the hight-T(P2/c) and the low-T (P/c) space groups of $Mo_8O_{23}$ . More rigorously, the group theory analysis in the high temperature phase shows that at $Z$ point of the Brillouin zone the spinor representations of the small group are four dimensional and as symmetrized square of this representations contains vector representation and the small group does not contain three fold and six fold axes, this point may have a Dirac crossing[3]. Indeed the Hamiltonian, constructed by the invariant technique shows Dirac nodal line. In 2D case single Dirac point would be present at $Z$[2], but for 3D the line of Dirac crossings appears along $k_y$ direction.

Further treatment of the low-temperature phase reduced symmetry demonstrates that the Dirac crossing is split in low temperature phase. The precise picture of splitting depends on details of theoretical treatment: while nodal Dirac loop around Z can be expected for a monolayer of $Mo_8O_{23}$ with broken inversion symmetry as in 2D limit[2] and from Hamiltonian with only linear-in-k invariants, GGA results demonstrate the clear splitting (see Fig. 3b in main text), although double degeneracy with Dirac dispersion still present at Z-point above and below the gap (cf. **Fig. S2**). Inclusion of higher-order invariants in the Hamiltonian could, in principle, reproduce this effect. However, GGA predicts decrease of the splitting from $\sim 100\,\mathrm{meV}$ down to $\sim 50\,\mathrm{meV}$ away from Z-point in directions other than $\Gamma - Z$, that is close to the ARPES resolution of $20\,\mathrm{meV}$ at $20\,\mathrm{K}$, and much less then the scale of bands smearing of $\sim 100\,\mathrm{meV}$ due to the correlation effects. Thus we cannot rule out that the large smearing simply obscures the splitting. But it is also possible, in principle, that the correlation effects prevent observation of the splitted Dirac crossing in more intricate way, similarly as they do this at energies close to the Fermi-level or that for some reasons one could regard surface (probed by STS and ARPES) as monolayer by symmetry.

## SUPPLEMENTARY METHOD 1: THE SHAPE OF TUNNELING SPECTRA AND EXTENDED DYNES FORMULA FIT

Simple DF analysis breaks down below $T \sim 175\,\mathrm{K}$ in estimating correctly the smearing, whereas the gap is reliably found to stay almost constant down to $40\,\mathrm{K}$ whereupon it starts to grow again, increasing 1.5 times at $9\,\mathrm{K}$. The simple

DF fit overestimates density of states at low energies, as shown in Fig. 6c of the main text. The deviation could be associated with the presence of two gaps in the spectrum. Indeed, since the multiband behavior could be expected from the transport and relaxation data, two gaps can coexist in the reciprocal space and add up in the tunneling spectra – the situation studied in details for CDW and Mott states in 1T-$TaS_2$[4] and for superconducting gaps in $MgB_2$[5, 6]. However, while the scenario is plausible, our data does not show any reliable signatures of the two-gap structure to start with (cf. Fig. 6c and **Fig. S3**). At the same time, fitting the data to the two-gap model involves five to seven parameters, none of which we are able to fix. We thus shall take a safer route and quantify the deviation with extended DF with only one additional parameter – a general energy-dependent term[7].

Tunneling selectivity could be responsible for preventing us from observing the two gaps simultaneously if they were present. Two gaps in STS were extensively studied in multiband superconductors, where two mechanisms were considered: pair[8] and inter-band quasiparticle[9] scattering. Both affect the density of states. However, the former fails to describe the present experimental data even when selectivity is taken into account, since here it implies the presence of two BCS-like gaps with sharp features in the tunneling spectrum[5, 6]. The quasiparticle scattering can produce smooth variation of the density of states inside the gap, but certain in-gap structure is still expected[10]. However the latter could also be revealed as the energy-dependent smearing and hence does not contradict the fit the data in our measurements. Thus, the two-gap scenario cannot be completely ruled out and orientation-resolved STS are necessary to elucidate this issue[6, 10].

We cannot directly interpret the physics beyond $\Gamma(E)$ dependence, though the energy dependence of a self-energy was suggested responsible for superconductors[7]. The latter suggests that electron-electron interactions could be important in this state, which makes it interesting to compare the low-T data with other correlated systems. Various Mott insulators are known to have characteristic power-law gaps[11], DOS $\propto (E - E_F)^\alpha$ with $\alpha \sim 1-2$. Interestingly, low-temperature tunneling spectra in $Mo_8O_{23}$ in double logarithmic scales show slopes with $\alpha$ changing from 1.5 to

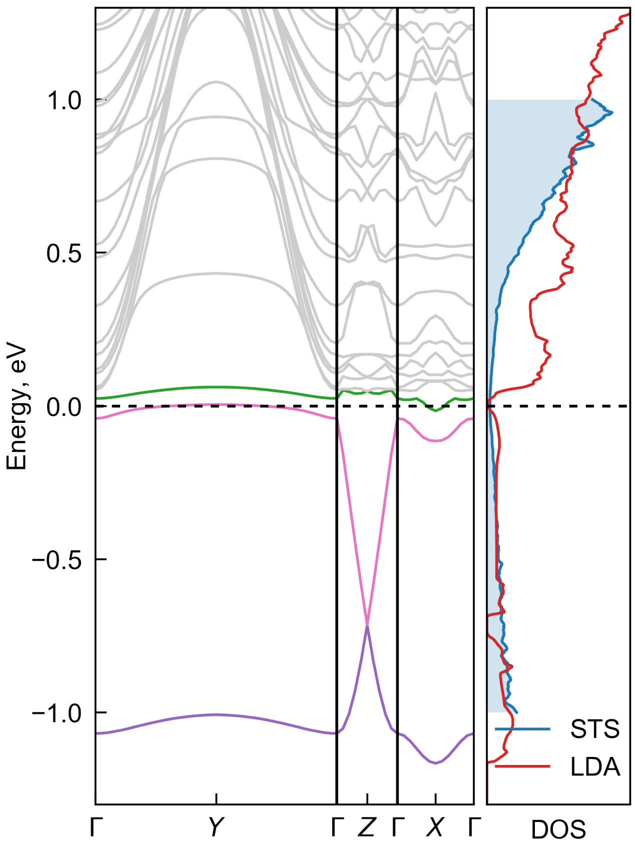


Figure **S1**. **Details of LDA calculations:** The two valence bands overlap with conduction bands in a semi-metallic manner with their top at +5 meV above the Fermi level and the bottom of the lowest conduction band that starts at −15 meV. This produces a small but finite DOS at the Fermi level. Right panel: LDA DOS is compared to the tunneling spectra (scaled).

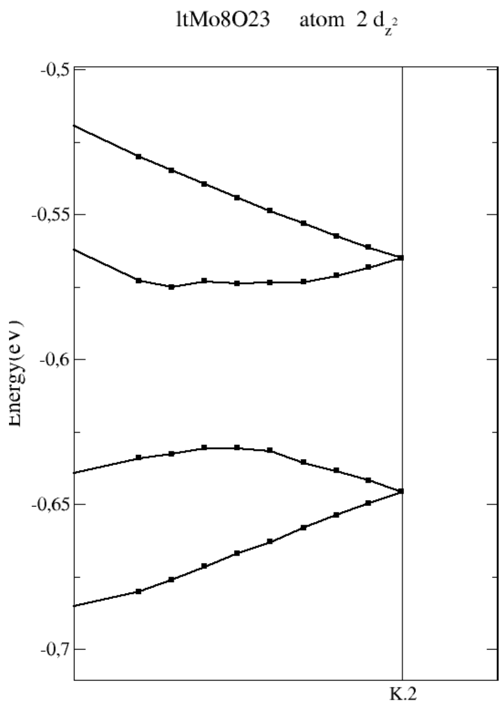


Figure **S2**. **Details of the Dirac crossing in the low-T band structure:**

2.5 as the gap opens (**Fig. S3**), which thus might indicate electronic correlations as the origin of the low-T state.

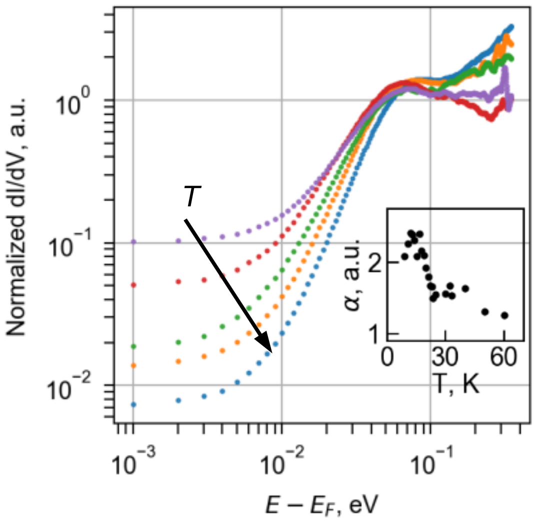


Figure **S3**. **Log-log plots of selected $dI/dV$ curves at low temperatures,** $T \in [9, 50]$ **K** The inset shows the temperature evolution of their slope $\alpha$ extracted in the $[10, 50]$ meV range.

## SUPPLEMENTARY DISCUSSION 2: MULTIPLE BANDS VS DFT RESULTS

While the evidence of multiple bands in $Mo_8O_{23}$ is unambiguous, the presence of a second electronic band crossing the Fermi level in the low-T phase is not predicted by DFT. Several scenarios can be put forward to explain this discrepancy. DFT shows the single electronic band at the Fermi level in the incommensurate phase that transforms into two (due to the doubling of the unit cell) electronic bands 25 meV above the Fermi level in the commensurate phase. The most intuitive scenario is that the band structure does not change abruptly at $T_{\mathrm{IC-C}} = 285\,\mathrm{K}$ but continuously evolves from high-T (see Fig. 3a in the main text) to low-T with increasing of CDW order parameter. The non-zero Grüneisen-like coefficients are then expected to drive the temperature evolution of the bands, yielding ungapped areas of Fermi surface. Note that the doubling of the unit cell along the b-axis, distinguishing the low-T and the high-T DFT band structures is present already in the incommensurate phase, favoring this scenario. Others rely on the correlation and orbital effects, which need to be investigated further.

## SUPPLEMENTARY DISCUSSION 3: NESTING SCENARIO

The origin of the high-T incommensurate CDW ordering in $Mo_8O_{23}$ is puzzling. As discussed in [12], one does not have a half-filled band and hence the low-temperature doubling of the unit cell along the b-direction is not expected. The incommensurate CDW ordering is also at first sight not expected, as the high-temperature structure is a semimetal (see Figs. 3, 4 in the main text). The influence of the quasi-1D physics would become more directly applicable for hole doping at the level $\approx 0.1$. Given the large unit cell, small non-stoichiometry per Mo would be sufficient to yield that. However, given the agreement of DFT with STS and given the Hall data that show small concentration of electrons and not holes this does not appear plausible. Another influence is that of the temperature dependence of the chemical potential due to the strong particle-hole asymmetry. At high temperatures the DFT chemical potential moves to the valence band. Similar effect can be expected from the large non-thermal smearing $\Gamma_0$ discussed above in the context of STS and ARPES data. One could envisage a possibility that electronic state would be strongly affected by the associated redistribution of electrons, which should be considered more carefully in future.

## SUPPLEMENTARY DISCUSSION 4: EXCITONIC INSULATOR SCENARIO

Unlike most of CDW compounds, $Mo_8O_{23}$ has semi-metallic rather than metallic band structure at high temperatures. The most well-known example of the CDW in the semi-metal is perhaps $TiSe_2$[13], where excitonic order has been also considered as a driving force for distortion[14–16]. The semi-metallic character of the DOS might explain the unusually weak dependence of the gap value on the temperature around $T_{\mathrm{ICDW}}$ in transient reflectivity measurements[17], as compared to the prototypical CDW compounds where high-T phase is metallic [18]. To understand the possible scenarios in such electron-hole system[19], it is important to know the relevant Fermi energy and band overlap. Hall effect measurements put lower bound on the electron concentration at the level of $\sim 10^{20}\,\mathrm{cm}^{-3}$ at $T \sim 200\,\mathrm{K}$. Using the calculated DOS (Fig. 3 in the main text), we obtain the estimate of Fermi level position with respect to the bottom of electronic band at $E_F - E_e \gtrsim 54\,\mathrm{meV}$, and, from the charge neutrality condition, for a hole band – at $E_h - E_F \gtrsim 55\,\mathrm{meV}$, giving the total overlap of $E_h - E_e \gtrsim 109\,\mathrm{meV}$. This value is comparable to the gap size $2\Delta \approx 100\,\mathrm{meV}$, and hence the excitonic insulator scenario for $Mo_8O_{23}$ cannot be ruled out[19].

* Venera.Nasretdinova@ijs.si


[1] *The mathematical theory of symmetry in solids* (Clarendon Press, 1972)
[2] S. M. Young and C. L. Kane, Phys. Rev. Lett. **115**, 126803 (Sep 2015)
[3] S. Yang, S. Zaheer, J. Teo, C. Kane, E. Mele, and A. Rappe, Phys. Rev. Lett. **108**, 140405 (2012)
[4] J.-J. Kim, W. Yamaguchi, T. Hasegawa, and K. Kitazawa, Phys. Rev. Lett. **73**, 2103 (1994)
[5] F. Giubileo, D. Roditchev, W. Sacks, R. Lamy, D. X. Thanh, J. Klein, S. Miraglia, D. Fruchart, J. Marcus, and P. Monod, Phys. Rev. Lett. **87**, 177008 (2001)
[6] M. Iavarone, G. Karapetrov, A. E. Koshelev, W. K. Kwok, G. W. Crabtree, D. G. Hinks, W. N. Kang, E.-M. Choi, H. J. Kim, H.-J. Kim, and S. I. Lee, Phys. Rev. Lett. **89**, 187002 (2002)
[7] R. C. Dynes, V. Narayanamurti, and J. P. Garno, Phys. Rev. Lett. **41**, 1509 (Nov 1978)
[8] H. Suhl, B. T. Matthias, and L. R. Walker, Phys. Rev. Lett. **3**, 552 (1959)

[9] W. L. McMillan, Phys. Rev. **175**, 537 (Nov 1968)
[10] Y. Noat, J. A. Silva-Guillén, T. Cren, V. Cherkez, C. Brun, S. Pons, F. Debontridder, D. Roditchev, W. Sacks, L. Cario, P. Ordejón, A. García, and E. Canadell, Phys. Rev. B **92**, 134510 (2015)
[11] Z. Wang, Y. Okada, J. O'Neal, W. Zhou, D. Walkup, C. Dhital, T. Hogan, P. Clancy, Y.-J. Kim, Y. F. Hu, L. H. Santos, S. D. Wilson, N. Trivedi, and V. Madhavan, Proc. Natl. Acad. Sci. **115**, 11198 (2018)
[12] E. Canadell and M.-H. Whangbo, Inorg. Chem. **29**, 2256 (1990)
[13] F. Weber, S. Rosenkranz, J.-P. Castellan, R. Osborn, G. Karapetrov, R. Hott, R. Heid, K.-P. Bohnen, and A. Alatas, Phys. Rev. Lett. **107**, 266401 (Dec 2011)
[14] M. P. an U. Leierseder, J.-M. Ménard, H. Dachraoui, L. Mouchliadis, I. E. Perakis, U. Heinzmann, J. Demsar, K. Rossnagel, and R. Huber, Nat. Mater. **13**, 857 (2014)
[15] Y. I. Joe, X. M. Chen, P. Ghaemi, K. D. Finkelstein, G. A. de la Peña, Y. Gan, J. C. T. Lee, S. Yuan, J. Geck, G. J. MacDougall, T. C. Chiang, S. L. Cooper, E. Fradkin, and P. Abbamonte, Nat. Phys. **10**, 421 (2014)
[16] A. Kogar, M. S. Rak, S. Vig, A. A. Husain, F. Flicker, Y. I. Joe, L. Venema, G. J. MacDougall, T. C. Chiang, E. Fradkin, J. van Wezel, and P. Abbamonte, Science **358**, 1314 (2017)
[17] V. Nasretdinova, M. Borovšak, J. Mravlje, P. Šutar, E. Goreshnik, T. Mertelj, and D. Mihailovic, Phys. Rev. B **99**, 085101 (2019)
[18] J. Demsar, K. Biljaković, and D. Mihailovic, Phys. Rev. Lett. **83**, 800 (1999)
[19] L. V. Keldysh, Contemporary Physics **27**, 395 (1986)
